# Supplementary material for: Comparative genomics provides new insights into the diversity, physiology, and sexuality of the only industrially exploited tremellomycete: Phaffia rhodozyma
Source: BMC Genomics. 2016 Nov 9;17:901. doi: 10.1186/s12864-016-3244-7 (PMC5103461; doi:10.1186/s12864-016-3244-7)
Supplement: Additional file 6: — List of orphan genes with links to PFAM (related to Additional file 1: Table S1). (ZIP 1428 kb) [file 12864_2016_3244_MOESM6_ESM.zip › PFAM_UNIQUE/G02422_P.html]

#                                                                            --- full sequence --- -------------- this domain -------------   hmm coord   ali coord   env coord
  
# target name        accession   tlen query name           accession   qlen   E-value  score  bias   #  of  c-Evalue  i-Evalue  score  bias  from    to  from    to  from    to  acc description of target
  
#------------------- ---------- ----- -------------------- ---------- ----- --------- ------ ----- --- --- --------- --------- ------ ----- ----- ----- ----- ----- ----- ----- ---- ---------------------
  
G02422\_P             -            657 DUF2454              PF10521.4    282     0.057   11.1   0.2   1   1     7e-05       0.1   10.2   0.2     7    58    82   132    78   145 0.89 - Protein of unknown function (DUF2454)  
G02422\_P             -            657 LRR\_5                PF13306.1    129     0.015   13.7   0.1   1   1   4.5e-06     0.027   12.9   0.1    69   102   322   355   309   368 0.48 - Leucine rich repeats (6 copies)  
G02422\_P             -            657 RRM\_1                PF00076.17    70   2.4e-05   22.5   0.0   1   2   1.5e-05    0.0012   17.2   0.0     1    68   120   182   120   184 0.90 - RNA recognition motif. (a.k.a. RRM, RBD, or RNP domain)  
G02422\_P             -            657 RRM\_1                PF00076.17    70   2.4e-05   22.5   0.0   2   2      0.41        31    3.0   0.0     5    21   200   216   197   221 0.88 - RNA recognition motif. (a.k.a. RRM, RBD, or RNP domain)  
G02422\_P             -            657 RRM\_6                PF14259.1     70   1.1e-05   24.0   0.1   1   2   5.5e-05    0.0042   15.7   0.0     1    69   120   183   120   184 0.92 - RNA recognition motif (a.k.a. RRM, RBD, or RNP domain)  
G02422\_P             -            657 RRM\_6                PF14259.1     70   1.1e-05   24.0   0.1   2   2     0.086       6.7    5.5   0.0     5    33   200   224   198   234 0.81 - RNA recognition motif (a.k.a. RRM, RBD, or RNP domain)
